# Transdiagnostic or Disorder Specific? Indicators of Substance and Behavioral Addictions Nominated by People with Lived Experience

**DOI:** 10.3390/jcm9020334

**Published:** 2020-01-24

**Authors:** Hyoun S. Kim, David C. Hodgins, Benjamin Kim, T. Cameron Wild

**Affiliations:** 1 Department of Psychology, University of Calgary, 2500 University Drive NW, Calgary, AB T2N 1N4, Canada; dhodgins@ucalgary.ca (D.C.H.); bekim@ucalgary.ca (B.K.); 2 School of Public Health, University of Alberta, 11405 87 Ave, Edmonton, AB T6G 1C9, Canada; cwild@ualberta.ca

**Keywords:** transdiagnostic, addictions, behavioral addictions, lay-epidemiology, lived experience

## Abstract

Using a transdiagnostic perspective, the present research examined the prominent indicators of substance (alcohol, cocaine, marijuana, tobacco) and behavioral (gambling, video games, sex, shopping, work, eating) addictions nominated by people with lived experiences. Specifically, we aimed to explore whether the perceived most important indicators nominated were consistent across the 10 addictions or differed based on the specific addiction. Additionally, we explored gender differences in the perceived most important indicators across addictive behaviors. A large online sample of adults recruited from a Canadian province (*n* = 3503) were asked to describe the most important signs or symptoms of problems with these substances and behaviors. Open-ended responses were analyzed among a subsample of 2603 respondents (*n* = 1562 in the past year) who disclosed that they had personally experienced a problem with at least one addiction listed above. Content analyses revealed that *dependence* (e.g., craving, impairments in control) and patterns of use (e.g., frequency) were the most commonly perceived indicators for both substance and behavioral addictions, accounting for over half of all the qualitative responses. Differences were also found between substance and behavioral addictions regarding the proportion of the most important signs nominated. Consistent with the syndrome model of addiction, unique indicators were also found for specific addictive behaviors, with the greatest proportion of unique indicators found for eating. Supplemental analyses found that perceived indicators across addictions were generally gender invariant. Results provide some support for a transdiagnostic conceptualization of substance and behavioral addictions. Implications for the study, prevention, and treatment of addictions are discussed.

## 1. Introduction

Transdiagnostic models of addictions are not new, having been proposed since the 1980s [1]. Since then, several transdiagnostic theories of addictions have emerged, including the excessive appetite model of addiction [2], the syndrome model of addictions [3], and the component model of addictions [4]. These transdiagnostic theories all propose that addictions, including behavioral addictions, should be defined by their similarities rather than their differences. In other words, rather than being conceptualized as distinct and unique disorders, addictions may be better viewed as representing a common underlying disorder with unique expressions (e.g., alcohol, cannabis, gambling, video games).

Consistent with a transdiagnostic conceptualization, a growing number of empirical studies suggest that the etiological (e.g., onset), neurobiological (e.g., reward pathway), phenomenological (e.g., loss of control, craving), psycho-social (e.g., impulsivity, interpersonal difficulties), and clinical (e.g., treatment strategies) features of substance and behavioral addictions overlap more often than not (see [5,6] for reviews). For example, both substance and behavioral addictions are neurobiologically associated with desensitization of reward circuits, dysfunctions in executive functions, and strengthening of conditioned addiction cues [7]. Regarding risk factors, childhood trauma has been associated with a variety of addictions, including alcohol, cannabis, food, and problematic Internet use [8]. Furthermore, psychological processes such as high levels of impulsivity and emotion dysregulation have also been robustly associated across both substance and behavioral addictions [6].

There exists, however, important differences regarding the behavioral components of substance addictions (compared to behavioral addictions). For example, the ingestion of psychoactive substances may lead to greater neurobiological effects (i.e., dopamine) for substance compared to behavioral addictions. Additionally, the physiological effects, including the intoxicating effects of substances may lead to unique and greater consequences of substance addictions compared to behavioral addictions. Indeed, some substances have greater physical dependency (e.g., opioids) and are associated with varying degrees of harms (e.g., alcohol), including potential for withdrawal symptoms [9]. The social environment, including the availability and acceptability of substance and behavioral addictions, may also be of importance for differences observed across addictions.

Having said that, the similarities between addictive disorders were recognized in the latest editions of the Diagnostic and Statistical Manual of Mental Disorders (DSM-5) [10] and the World Health Organization’s International Classification of Diseases (ICD-11). Specifically, pathological gambling, renamed gambling disorder, was moved from the Impulse Control Disorder section and joined psychoactive substances in the Substance-related and Addictive Disorders section, becoming the first behavioral addiction to be recognized as such [11]. Internet gaming disorder was included in Section 3 of the DSM-5 as a disorder requiring further empirical study. In contrast, the ICD-11 included both gambling disorder and gaming disorder alongside psychoactive substances as a disorder due to the addictive behaviors section of the manual [12]. Moreover, the diagnostic criteria across both substance and behavioral addictions are nearly identical in the DSM-5 and in the ICD-11, further supporting a transdiagnostic conceptualization of addictions.

Despite the growing interest in examining addictive disorders from a transdiagnostic perspective [13], empirical studies testing transdiagnostic theories of addictions are rare. A recent systematic review found a lack of empirical studies investigating transdiagnostic concepts. This was particularly true for the field of addictions [14]. Furthermore, the few transdiagnostic studies of addictions have compared only a handful (i.e., 2–3) of addictive behaviors (see [15] for a recent review of behavioral addictions). These studies have generally concluded that different expressions of addictive behaviors (e.g., alcohol, gambling, gaming) overlap in neurobiological, personality, and clinical characteristics. However, in line with more recent transdiagnostic models [3,6], addictive behaviors also present with unique differences that may have important etiological and treatment implications. Extending these findings, Shaffer and colleagues [16] investigated the demographic and psychological characteristic of people seeking treatment for a variety of substance (e.g., alcohol, stimulants) and behavioral (e.g., gaming, compulsive sexual behavior) addictions. The authors found more similarities (e.g., depression, impulsivity) than differences (e.g., state anxiety) between people seeking treatment for substance and behavioral addictions, lending support for a transdiagnostic conceptualization of addictions. While informative, these studies are limited by their small sample sizes. Furthermore, the aforementioned studies consisted of treatment-seeking samples, which may provide a limited understanding of the common characteristics of addictions given that the rates of treatment seeking remain low for both substance and behavioral addictions [17,18].

In addition to the limited empirical studies investigating the transdiagnostic theories of addictions, the study of behavioral addictions in general has been criticized on numerous grounds [19]. First and foremost, it has been argued that atheoretical and confirmatory approaches used by researchers to identify disorders have led to the creation of dozens of “new” behavioral addictions, resulting in the over pathologizing of daily life activities and potentially reducing the relevance and credibility of the field of addictive disorders as a whole [19]. More specifically, substance use disorder diagnostic criteria are often applied to other behaviors without careful determination of their suitability. For example, diagnostic criteria for behavioral addictions have included tolerance and withdrawal symptoms, which may not necessarily apply to behavioral addictions [20]. Furthermore, putative behavioral addictions have been proposed without a careful examination of the associated harms, which may differ from substances [21].

In response to the above concerns, a person-centered qualitative approach examining the characteristics of behavioral addictions from those with lived experiences has been recommended as a way to improve the current methodological limitations in the study of behavioral addictions [21]. It has also been suggested that the study of potential behavioral addictions should be theory driven, which is in contrast to the current atheoretical approaches [21]. Despite these recommendations, such methodological approaches are rarely used in the field of addictive disorders generally and in the field of behavioral addictions specifically.

To address these empirical gaps, the present study collected first-person indicators of the most important signs and symptoms of 10 addictive behaviors; four substances (alcohol, tobacco, marijuana, cocaine) and six behaviors (gambling, shopping, video gaming, eating, sex, and work) from people with lived experiences. The aim of the present research was to examine whether the most important indicators nominated by people with lived experiences were consistent across the 10 addictions or differed based on the specific addiction. We also conducted exploratory analyses examining whether men and women report differences in the most important indicators across the 10 addictive behaviors. We know, for example, while the indicators for gambling tend to be generally similar for men and women, men with gambling problems are more likely to report aggressive behavior towards gambling devices, whereas women are more likely to report indicators of emotional distress [22]. However, it is currently unknown whether gender differences also exist in the indicators for other addictive behaviors.

In the present research, we adopted an inductive, lay epidemiology approach to identify the most important indicators of problematic involvement in the 10 addictive behaviors. Lay epidemiology proposes that, “…fields of symptomatology, nosology, aetiology, and epidemiology have identifiable counterparts in the thoughts and activities of people outside the formal medical community” [23]. As such, a lay epidemiological perspective counterbalances the traditional expert-derived conception of signs and symptoms of addictive behaviors and has provided valuable insight in the field of addiction. For example, a lay epidemiologic analysis of drinkers in the UK demonstrated that they generally disregard current low-risk drinking guidelines derived from experts because the criteria do not address drinkers’ own conceptions of what would count as an alcohol problem [24]. More recently, a lay epidemiological approach was used to develop a unified screener of substance and behavioral addictions. Through factor analyses, Schluter et al. [25] found that from an initial pool of 15 items, four items demonstrated excellent psychometric properties in screening for different addictions, providing a comprehensive tool to screen for various addictions.

## 2. Methods

### 2.1. Participants and Procedure

Using procedures previously employed to systematically describe lay epidemiologic views on addictive behaviors [26,27], a third-party firm was contracted to conduct 3503 online surveys in November and December of 2013, drawing on an online panel of respondents aged 18 or older. Randomly sampled men and women from all regions of Alberta were invited to participate. No area or gender-based quotas were set for completed interviews. Rather, a quota was set for 3500 completed responses. Randomly sampled participants received invitations to take part in the survey, which was described as an opportunity to answer “…questions about your experience of, and beliefs about, different kinds of addictive behaviors”. Each email invitation included a survey URL, embedded with a personal identification number. A total of 6721 panel members accessed the survey with 3503 completing the survey, which represents a response rate of 61.6%. Respondents completed the survey at their own convenience. Two steps were taken in order to maximize participation and mitigate non-response bias: (1) email reminders to complete the survey were sent approximately one week and two weeks after initial invitation and (2) an incentive was provided to all those who completed the survey. The sample size of 3503 produced parameter estimates accurate to within ±1.7 percentage points, 19 times out of 20, of what they would have been had the entire population of Albertans 18 years or older been surveyed.

### 2.2. Identifying Respondents with Lived Experience

Participants were initially asked to report on their personal experiences with each of the 10 target behaviors of interest (alcohol, tobacco, marijuana, cocaine, gambling, shopping, video gaming, eating, sex, and work); “Thinking back over your life, have you ever personally had a problem with [problem behaviors]?” Response options were no, yes (in the past 12 months), yes (but not in the past 12 months), don’t know, and prefer not to say. Participants who indicated yes (either in the past year or otherwise) for one or more of the 10 target substances and behaviors were coded as respondents with lived experience and comprised the analytic subsample for this study.

### 2.3. Eliciting Indicators of Problematic Substance Use and Behavioral Engagement

All participants were then randomly assigned by a computer algorithm to answer questions about one of the 10 target substances or behaviors. “What sign or symptom is most important in showing that someone has a problem with [randomly-assigned substance or behavior]?” and “What sign or symptom is most important in showing that someone is a/an [randomly assigned substance or behavior] addict?” Responses to both questions were combined to provide a pool of indicators describing the most important features or characteristics of addictions from the perspective of people who reported a past-year or lifetime problem with one or more of the 10 target substances and behaviors. In order to obtain indicators from as many people with first-hand experience as possible, the elicitation questions examining the most important indicators were asked for up to three additional addictions that a participant reported having personally experienced. If participants indicated four or more, they were asked to report the most important sign/symptom for up to three. Participants were requested to be detailed and specific in their responses, which they typed in the space provided.

### 2.4. Data Analytic Plan

Thematic content analysis was used to identify themes and patterns from the open-ended responses to derive fewer categories with the same meaning or content [28]. First, participants’ responses were parsed into codable “meaning units,” each expressing a single concept or idea [29] using NVivo 12. This data reduction process resulted in 12,769 codable meaning units. Consistent with a lay-epidemiological approach, an inductive approach was initially used to develop a categorical coding scheme for each addiction to identify the perceived most important signs and symptoms. Next, a deductive approach was used to condense the large number of identified categories into main and subcategories (Table 1). This process was developed iteratively and was guided by the empirical literature. For example, a main category (*dependence*) was created that reflected DSM-5 criteria of addiction as well as symptoms that were purported to be hallmark characteristics of addictions such as prioritization and coping [4]. A decision was made to separate out harms as it was suggested that the identification of harms associated with behavioral addictions may help to conceptualize behavioral addictions without pathologizing common everyday behaviors [21]. Lastly, the frequency of responses was also taken into account when developing the categories. For example, unique indicators that did not have a sufficient number of responses to warrant consideration as a main category were then categorized as subcategory.

Responses were classified as “uncodable” if they were unintelligible, vague, unclear, blank, or nonsense. Two independent raters coded all the responses. The reliability of the coding scheme was examined by multiple independent raters who coded a randomly drawn set of 15% of responses from each addiction for a total of 2196 responses. The kappa statistic between the coders was 0.31. However, kappa statistics can be low in situations where the percentage agreement is high and the percentage disagreement is low, which is known as the kappa paradox [30]. This was the case in the present research, in which the overall percentage agreement across the coders was 99.4%. Discrepancies were resolved through consensus between the reviewers and the first author.

Mann–Whitney U and chi-squares were conducted to examine demographic differences between participants who reported a first-hand experience with addictions in the past 12 months, lifetime experience with addictions, and to those without a first-hand experience. Descriptive statistics were used to assess the frequency and proportion of the most important indicators of the 10 addictive behaviors. Chi-square analyses were conducted on the main categories across the 10 addictions to examine whether specific indicators (e.g., dependence, patterns of use, impairments) were more likely to be reported for certain addictions. Next, we collapsed the responses between substance and behavioral addictions to assess whether substance and behavioral addictions differed regarding the most important indicators. Lastly, chi-square analyses were used to examine whether men and women reported differences regarding codes reflecting the most important signs/symptoms across the 10 addictive behaviors.

## 3. Results

Of the total sample of 3503, 2603 (74.3%) reported that they had experienced a past-year or lifetime problem with one of the ten addictions. Of the 2603, 1562 reported experiencing a current problem. Furthermore, 1009 reported experiencing current or lifetime problem with one of the 10 target substances and behaviors, 807 with two, and 787 with three or more. The most commonly reported first-hand problem with an addiction was tobacco (*n* = 1353), followed by eating (*n* = 1125), work (*n* = 968), alcohol (*n* = 662), sex (*n* = 364), shopping (*n* = 324), cannabis (*n* = 264), gambling (*n* = 254), video games (*n* = 236), and cocaine (*n* = 130). The 2603 respondents who indicated a first-hand problem with addictions provided a total of 9956 responses, resulting in 12,769 codable meaning unit. Tobacco had the greater number of codable meaning units (*n* = 3106), followed by eating (*n* = 2486), work, (*n* = 2119), alcohol (*n* = 1571), sex (*n* = 784), shopping (*n* = 722), video games (*n* = 582), cannabis (*n* = 558), gambling (*n* = 545), and cocaine (*n* = 296).

**Figure 1:**
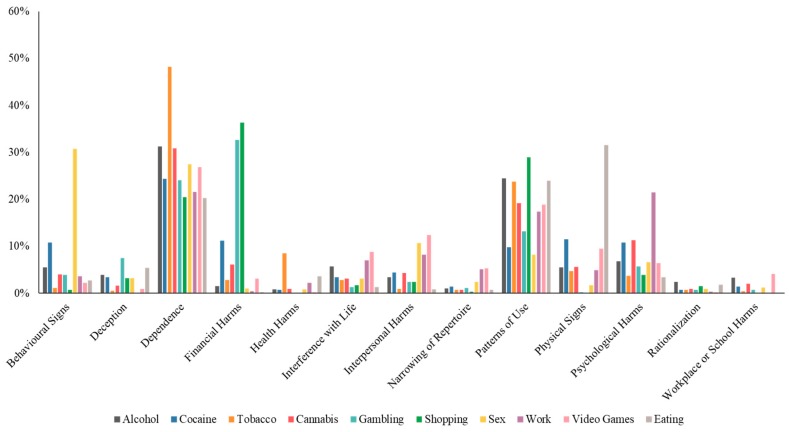
Percentage of responses for the perceived most important indicators across the 10 addictive behaviors.

There were significant demographic differences between participants who indicated a current experience with addictions, past experience with addictions, or with no first-hand experience with addictions. Specifically, participants who reported currently experiencing a problem with an addiction were younger, more likely to be single, unemployed, and have lower levels of education (Table 2). Overall, *dependence* (30.1%) and *patterns of use* (21.0%) were the two categories that were perceived as the greatest indicators across addictive behaviors, accounting for over half of all the qualitative responses (Figure 1). The *dependence* theme was characterized by descriptions that aligned with the DSM-5 conceptualization of addictions including craving, impairment of control, preoccupation, tolerance and withdrawal as well as other characteristics that are purported to be hallmark symptoms of addictions such as coping and preoccupation [4]. *Patterns of use* included reference to how someone engaged in the behavior in terms of quantity, style or timing of use.

*Dependence* was also perceived as the greatest indicator for six of the ten addictions, with the exceptions being gambling (*financial harms*), shopping (*financial harms*), sex (*behavioral signs*), and eating (*physical signs*). *Dependence* and *patterns of use* were followed by *physical signs* (9.8%), *psychological harms* (7.9%), *financial harms* (5.2%), *behavioral signs* (4.6%), *interpersonal harms* (3.8%), *interferes with life* (3.7%), *health harms* (3.3%), *deception* (2.6%), *narrowing of repertoire* (1.8%), *rationalization* (1.1%), and *workplace/school harms* (0.9%). 2.9% of the total responses were coded as *don’t know* and 1.3% of the responses were coded as *other* (Table 3) (Figure 1).

There were significant differences across the addictive behaviors in the perceived most important indicator. Behavioral signs were the most commonly reported for sexual addiction (30.7%), deception was the most commonly reported for gambling (7.5%), dependence for tobacco (48.2%), physical signs for eating (31.5%), rationalization for alcohol (2.4%), financial harms (36.3%) and patterns of use (29.0%) for shopping, interfering with life (8.8%), interpersonal harms (12.4%), narrowing of repertoire (5.3%) and workplace/school harms (4.1%) for video games, health harms for tobacco (8.4%), and psychological harms for work (21.4%).

When collapsing across substance and behavioral addictions, dependence and patterns of use had the highest proportion of responses regarding the perceived most important indicator for both substance and behavioral addictions (Table 4). However, differences emerged with dependence, health harms, patterns of use, and workplace/school harms being more likely to be reported for substances, whereas behavioral signs, deception, financial harms, interpersonal harms, narrowing of repertoire, physical signs, and psychological harms were more commonly reported for behavioral addictions. No statistically significant differences were found for interferes with life and rationalization between substance and behavioral addictions.

Nine of the 10 addictive behaviors were associated with unique indicators as nominated by people with lived experiences (Table 5). No unique indicators were described for excessive work involvement. Eating had the greatest proportion of unique indicators, which included *health harms* (e.g., diabetes) as well as *maladaptive eating patterns* (e.g., eating without hunger). Several unique indicators found were attributed to the physiological effects of the addictive behaviors, such as *hangovers* and *motor coordination* for alcohol, *runny or red nose* for cocaine, and *yellowing of finger and nails* for tobacco use. Unique indicators were also found for behavioral addictions, including *playing all night* and *unreality* (i.e., difficulty separating real life and fantasy) for video games, *promiscuity/indiscriminate behavior*, *relationships difficulties* including *infidelity*, *erection problems* for sex, *hoarding and clutter, unnecessary purchases for shopping*, and *chasing losses* for gambling.

Overall, the perceived most important indicators of the addictive behaviors were invariant across genders, with only few significant differences between men and women (see Supplementary Tables). Tobacco had the greatest differences in regard to the reported indicators by men and women, with men more likely to identify health harms, patterns of use, physical signs, and workplace/school harms as the perceived most important indicator of tobacco problems, whereas women were more likely to nominate signs of dependence. Significant differences were also found for eating with women more likely to perceive deception, dependence, and interpersonal harms as the most important indicators, whereas men were more likely to report physical signs. Regarding sex, men were more likely to report deception and women were more likely to report psychological harms as being the perceived most important indicator. For cannabis, women were significantly more likely to report psychological harms. Women were also more likely to report financial harms as the perceived most important indicator for gambling and psychological harms for work. No gender differences were found for alcohol, cocaine, shopping, or video games.

## 4. Discussion

There is a growing interest in transdiagnostic conceptualizations of psychiatric disorders, including addictions. This interest may stem in part from the symptom overlap and multiple diagnostic categories that exist in the current diagnostic categories [31] and questions regarding the clinical utility of the current diagnostic system [32]. Yet, empirical studies testing transdiagnostic conceptualizations of disorders in general are rare, particularly in the addiction field [14]. The aim of the present research was to address this gap by describing lay accounts of the most important indicators of problems with 10 different substances and behaviors from people who reported that they had personally experienced a problem with one or more of them. Specifically, we aimed to examine whether the most important indicators nominated by people with lived experiences were consistent across the 10 addictions or differed based on the specific addiction. To this end, we employed a person-centered qualitative approach as it has been suggested that such methodology is an important step in addressing the current limitations that exist in the empirical study of behavioral addictions.

Of the total sample, 74% reported experiencing a life-time problem with at least one of the ten addictive behaviors, with 44.59% reporting currently experiencing a problem with an addiction. This finding is in line with a review that suggests that 47% of the adult US population may present with problems with an addictive behavior within a 12-month period [33]. In regard to specific addictive behaviors, the most commonly reported problems were with tobacco and with excessive involvement in everyday behaviors such as work and eating. The finding that people were most likely to report personally experiencing problems with tobacco is not surprising given tobacco use remains relatively high despite the significant decline in the past 50 years [34]. A relatively large proportion of participants also reported experiencing problems with everyday behaviors such as eating and work. This finding is in need of further investigation given that although behavioral addictions are among the more common psychological disorders [35], they currently lack well-developed diagnostic criteria assessments. Indeed, the word “addiction” is a loaded term and may be used to describe a bad habit or in marketing for products (e.g., video games). As such, the rates of people reporting a personal problem with addictive behaviors found in the present research may be elevated.

Overall, the most important indicators reported by people with lived experiences were dependence and patterns of use, which accounted for over half of the codable responses. Dependence was also the most commonly reported symptom for six of the ten addictions, and of the four others, signs of dependence was among the top three most commonly nominated signs/symptoms. The dependence category aligns with the current DSM-5 criteria for addictive disorders and the patterns of use generally referred to excessive engagement. As such, the result of the present research provides some confidence in the current diagnostic criteria and hallmark characteristics for addictive behaviors. Additionally, the results suggest similar indicators may also apply to behavioral addictions and may aid in the development of diagnostic criteria for emerging behavioral addictions, including everyday behaviors such as work, sex, shopping, and eating. In other words, the results provide some support for the creation of transdiagnostic criteria for addictive disorders in general, regardless of the different manifestations (e.g., alcohol, cannabis, gambling, video games). Indeed, although not explicitly stated as such, the diagnostic criteria for addictive disorders in the DSM-5 can be seen as transdiagnostic in nature given the significant overlap in the criterion across the substance and behavioral (gambling, Internet gaming) addictions.

Having said that, there were differences regarding the proportion of the most important indicators across the addictive behaviors, including when the substances and behaviors were collapsed. These results suggest that while addictive behaviors may manifest similarly, some indicators may be more pertinent to one addiction over another. For example, financial harms were most frequent for gambling and shopping, suggesting this indicator may have high specificity in the diagnosis of these two addictive behaviors, compared to work and eating, in which the proportion of these responses was less than 1%. Additionally, significant differences were found regarding the most important indictors between substance and behavioral addictions. For example, while dependence was the most common indicator for both substances and behaviors, people with lived experiences were two times more likely to note dependence as an indicator for substances than behavioral addictions. A potential reason for this difference could be due to greater neurobiological impact of substances and symptoms of withdrawal and tolerance, which as previously mentioned may not be applicable to behavioral addictions. The psychoactive mechanism associated with substances may also have resulted in health harms being more likely to be nominated as an important indicator for substance addictions. In contrast, financial, interpersonal, and psychological harms were more likely to be nominated as important indicators for behavioral addictions. This finding may be due to the lack of “overdose” for behavioral addictions. In other words, it is possible to engage in behavioral addictions such as gambling, gaming, and shopping for a significantly longer period of time than using substances, which may lead to more financial harms (e.g., gambling, shopping), interpersonal harms (e.g., gaming) and psychological harms. Taken together, the differences in the most important indicators for substance and behavioral addictions may not support a transdiagnostic understanding of addictions. Furthermore, the aforementioned differences between substance and behavioral addictions should be taken into account in the conceptualization and treatment of addictive behaviors

As anticipated, nine out of ten addictive behaviors also were described with unique indicators, which is in line with the syndrome model of addictions [3]. Several of the unique indicators were a result of the physiological effects of the addictive behavior, such as hangovers for alcohol, runny/red nose for cocaine, and yellowing of fingers and nails for tobacco. In regard to behavioral addictions, behavioral signs related to the addictive behavior were found to be unique, for example, indiscriminate behaviors for sex, chasing losses for gambling, and unhealthy eating habits for eating. Given that people may be hesitant to self-report symptoms of addictions due to stigma, observing for these physical and behavioral signs along with validated methods (e.g., structured clinical interviews, self-report scales) may assist clinicians in diagnosis of addictions. Several of the unique indicators were related to frequent mental health co-morbidities such as insomnia for video games [36] and hoarding for shopping [37]. These results suggest that although the indicators of addictions may generally overlap, addictive behaviors may nevertheless present with unique psychological co-morbidities. Having said that, it is important to keep in mind that people with lived experiences nominated what they perceived to be the most important indicator of the addictions. As such, the results do not truly reflect whether a nominated sign or symptom (e.g., long term use) applies or does not apply to other addictive behaviors. Rather, the unique indicators reflect perceived most important indicators that were nominated for one addiction but not others.

Similar to previous research [22], we found that the indicators of the different addictive behaviors were generally gender invariant. Indeed, no differences were found in how men and women described the most important indicators for alcohol, cocaine, shopping, and video games, whereas cannabis/work (psychological harms) and gambling (financial harms) presented with differences in only one domain. The greatest proportion of gender differences was found for tobacco and eating. It is unclear as to why tobacco and eating would present with the greatest gender differences at the present moment. Thus, future research replicating our findings would be highly informative. Interestingly, when gender differences were found in regard to psychological harms (cannabis, work, and sex), women were significantly more likely to report this domain as an important indicator compared to men. This finding is in line with Delfabbro and colleagues [22] and suggests women may view signs of distress (e.g., depression, anxiety, stress) as a key indicator of an addiction.

Taken together, the results of the present research provide some support for a transdiagnostic conceptualization of addictions, which may have important clinical implications. For example, given that the signs of different addictions tend to be more similar than they are different, it may be more efficient to utilize unified assessment measures [25] to assess for a wide array of addictive behaviors rather than employing multiple measures. Such an approach would not only reduce fatigue and burden given people would only need to complete one single questionnaire rather than dozens of measures, it also has the added benefit of assessing the same constructs across addictive behaviors, which helps to provide more reliable prevalence rates and comparisons. Regarding treatment implications, a development of transdiagnostic treatment for addictions that targets the hallmark symptoms and common mechanisms of addictions (e.g., impulsivity, emotion dysregulation) may be beneficial. Indeed, a transdiagnostic treatment of addictions may be more efficient in addressing co-occurring addictions and may also minimize addiction substitution compared to disorder specific protocols [6]. With that said, some of our results did not support a transdiagnostic conceptualization of addictions, with significant differences found in the indicators nominated across the 10 addictions as well as between substance and behavioral addictions. These differences would need to be taken into account in the conceptualization, prevention, and treatment of substance and behavioral addictions.

## 5. Limitations

The results of the present research could have been complimented with the use of clinical interviews and instruments to verify participants’ experience of addictions. Having said that, we asked participants to self-indicate whether they had personal experience as consistent with the lay epidemiology perspective. Furthermore, studies have found that individuals tend to be able to accurately self-identify having problems with addictive disorders [38]. Second, the way the questions were asked should be noted as a limitation. Specifically, we asked people with lived experiences to nominate the most important signs and symptoms of addictions. That is, the question was not meant to elicit all possible important signs and symptoms of addictions. It is likely that had the question been asked of participants to nominate the signs and symptoms of addictions (rather than most important), people with lived experiences may have provided different answers. Related to the above, how we defined the 10 addictions should be noted as a limitation. That is, we did not provide a definition of addictive disorders as we did not want to influence participants’ perceptions. The framing of the question may have influenced participants responses, especially in regard to everyday behaviors such as eating. For example, the perceived most important indicator for eating may be related to eating disorders, as opposed to eating addiction. As such, care should be taken when interpreting our results to everyday behaviors. Fourth, there were differences regarding the representative nature of our sample compared to the 2011 Albertan census. For example, the composition of our sample was older and included more women compared to the Albertan population. Indeed, the majority of responses collected in the present study came from middle-aged adults. In contrast, only a small number of signs and symptoms were elicited from Albertans aged 18–34 years old. Thus, the results of the study may not extend to other age groups such as youths and older adults who may have different perceptions regarding the most important signs and symptoms of addictive behaviors. Relatedly, our sample consisted of more women than men, which could have biased the results. With that said, the most important indicators of addictions nominated by people with lived experiences were largely gender invariant. Fifth, the fact that participants required Internet access should be noted as a limitation. Although the majority of people have access to the Internet, there may be important differences regarding age, socioeconomic status, and location between people who do and do not have access to the Internet. Indeed, the fact that participants needed access to the Internet to participate in the present research may have resulted in them being highly educated.

## 6. Conclusions

Addictive disorders are one of the most common psychological disorders in society [32]. The concept of addictive disorders has also expanded with the emergence of behavioral addictions, including everyday behaviors. Herein, we investigated whether addictive disorders could be conceptualized from a transdiagnostic perspective by examining the most important indicators of 10 addictive behaviors from the perspective of people with lived experiences. The results compliment the prevalent top-down approach to the study of addictions and provides preliminary support for a transdiagnostic conceptualization of addictions, which may have important implications in the study, prevention, and treatment of addictions.

## Figures and Tables

**Table 1:** Coding scheme consisting of main and subcategories for the 10 addictive behaviors.

Main Category	Subcategories
**Behavioral Signs (4.7%)**: Behaviors that point to issues or signs of a problem.	Change in behavior (differences…after introduction of substance/behavior) Risky and illegal activities (i.e., stealing, simultaneous substance use) Masturbation (sex) Pornography and Internet use (sex) Promiscuity and indiscriminate behavior (sex) Snacking (eating)
**Deception (2.6%)**: Engaging in efforts to hide addiction from others.	(*no subcategories*)
**Dependence (30.1%)**: Signs and symptoms that reflect DSM-5 conceptualization of addictions and purported hallmark symptoms of addictions.	Coping (use of behavior or substance to relieve or decrease negative affect) Craving (wanting or feeling an urge to use the substance) Impaired control (an inability to say no to the behavior or substance) Need to function (Being unable to act normally or do certain things without the substance) Preoccupation (obsession, always talking /thinking about it) Prioritization (putting substance/behavior explicitly over other people/activities) Tolerance Withdrawal
**Financial Harms (5.2%)**: Financial difficulties as a result of addiction.	Borrowing money Credit card misuse Lack of money Overspending Selling items
**Health Harms (3.3%)**: Harms to health and well-being.	Coughing and lung issues (tobacco and…cannabis) Diabetes (eating) Digestive issues (eating) High cholesterol (eating)
**Interferes with Life (3.7%)**: Addiction starts to affect daily living.	(*no subcategories*)
**Interpersonal Harms (3.8%)**: Hurting relationships with others.	Changing social circle Social withdrawal Concern from others Romantic problems (sex)
**Narrowing of Repertoire (1.8%)**: When life becomes all about the addiction.	(*no subcategories*)
**Patterns of Use (21.0%)**: Qualities or descriptions of how someone engages in the addiction.	Any use Binge use Excessive engagement Intrinsic value Unusual times Chasing losses (gambling) Abnormal eating habits (eating) Hoarding/clutter (shopping) Unnecessary purchases (shopping)
**Physical Signs (9.8%)**: Physical signs that point to problems with substance or behavior.	Eating signs (all except eating) Eye signs Hangovers (alcohol) Impaired motor coordination Nose issues (cocaine, alcohol) Passing out (alcohol) Sleeping or fatigue signs Smell Speech impairment (alcohol) Weight signs Nicotine or yellowing of fingers (tobacco) Erectile problems (sex) Difficulty having sex (sex)
**Psychological Harms (7.9%)**: Negative psychological consequences.	Anhedonia Cognitive harms (lack of focus, memory loss) Mood swings Fulfillment Negative affect (depression, irritability, anger, stress and anxiety) Personality changes Regret Self-esteem and self-worth
**Rationalization (1.1%)**: Lack of awareness that one has an addiction or resistance to the idea that there is an addiction.	Denial or lack of awareness
**Workplace or School Harms (0.9%)**: Negative consequences to professional life or wellbeing.	(*no subcategories*)
**Don’t Know (2.9%)**: Participants reporting they do not know or are unsure about the most important symptom of addiction	(*no subcategories*)
**Other (1.3%)**: Responses that are not categorizable into any existing categories, but are not considered to be uncodable	(*no subcategories*)
**Uncodable**: Vague, unclear, blank, or nonsense, responses	(*no subcategories*)

Brackets denote that this subcategory was only applicable to the specific addiction.

**Table 2:** Comparison of demographic characteristics between participants with and without personal experiences with the 10 addictive behaviors.

	No Personal Experience with Addiction (*n* = 900)			Past 12-Month (Current) Personal Experience with Addiction (*n* = 1562)			Lifetime Personal Experience with Addiction (*n* = 1041)			Test	*p*
**Characteristics**	*N*	%	*M* (*SD*)	*N*	%	*M* (*SD*)	N	%	*M* (*SD*)		
**Age**			49.8 (15.0)			47.7 (13.8)			52.3 (14.8)	*F* = 31.39	**<0.001**
**Gender**										*χ^2^* =4.80	0.910
Male	378	42.1		647	41.1		475	45.6			
Female	520	57.9		915	58.6		566	54.4			
**Marital Status**										*χ^2^* = 19.45	**<0.001**
Married/Stable partner	617	68.7		944	60.4		696	66.9			
Single	277	30.8		604	38.7		340	32.7			
**Employment Status**										*χ^2^* = 98.48	**<0.001**
Full time employment	452	50.3		814	52.1		483	46.4			
Part time employment	112	12.5		164	10.5		116	11.1			
Unemployed	37	4.1		97	6.2		38	3.7			
Student	23	2.6		53	3.4		28	2.7			
Retired	198	22.0		261	16.7		306	29.4			
Not working due to disability	18	2.0		93	6.0		26	2.5			
Other	52	5.8		70	4.5		39	3.7			
**Education**										*χ^2^* = 49.09	**<0.001**
Less than high school	29	3.2		86	5.5		36	3.5			
High school	121	13.5		266	17.0		160	15.4			
Some university	157	17.5		395	25.3		240	23.1			
University undergraduate degree/college technical diploma	467	52.0		657	42.1		471	45.2			
University graduate or professional degree	119	13.3		155	9.9		133	12.8			

**Note.** Bold denotes significant differences.

**Table 3:** Percentage of responses and differences regarding the perceived most important indicators between the 10 addictive behaviors.

	Alcohol	Cocaine	Tobacco	Cannabis	Gambling	Shopping	Sex	Work	Video Games	Eating	Total
Dependence	31.3%	24.3%	**48.2%**	30.8%	24.0%	20.5%	27.4%	21.5%	26.8%	20.2%	30.1%
Patterns of Use	24.4%	9.8%	23.8%	19.2%	13.2%	**29.0%**	8.2%	17.4%	18.9%	24.0%	21.0%
Physical Signs	5.5%	11.5%	4.7%	5.6%	0.2%	0.0%	1.7%	4.9%	9.5%	**31.5%**	9.8%
Psychological Harms	6.8%	10.8%	3.7%	11.3%	5.7%	3.9%	6.6%	**21.4%**	6.4%	3.4%	7.9%
Financial Harms	1.5%	11.2%	2.8%	6.1%	32.7%	**36.3%**	1.0%	0.4%	3.1%	0.2%	5.2%
Behavioral Signs	5.5%	10.8%	1.1%	3.9%	3.9%	0.7%	**30.7%**	3.5%	2.2%	2.7%	4.7%
Interpersonal Harms	3.4%	4.4%	0.9%	4.3%	2.4%	1.0%	10.7%	8.2%	**12.4%**	0.8%	3.8%
Interferes with Life	5.7%	3.4%	2.8%	3.1%	1.3%	1.7%	3.1%	7.0%	**8.8%**	1.3%	3.7%
Health Harms	0.8%	0.7%	**8.4%**	0.9%	0.0%	0.0%	0.8%	2.2%	0.2%	3.6%	3.3%
Deception	3.9%	3.4%	0.5%	1.6%	**7.5%**	3.2%	3.2%	0.1%	0.9%	5.4%	2.6%
Narrowing of Repertoire	1.0%	1.4%	0.7%	0.7%	1.1%	0.3%	2.4%	5.1%	**5.3%**	0.7%	1.8%
Rationalization	**2.4%**	0.7%	0.7%	0.9%	0.7%	1.5%	0.9%	0.3%	0.2%	1.8%	1.1%
Workplace or School Harms	3.3%	1.4%	0.4%	2.0%	0.7%	0.1%	1.2%	0.0%	**4.1%**	0.1%	0.9%

**Note.** Bold denotes behavior with the greatest percentage of responses for that category.

**Table 4:** Percentage of responses and differences in theeq perceived most important indicators collapsed across substance and behavioral addictions.

	Substances	Behaviors	χ^2^	*p*
Behavioral Signs	3.2%	**5.8%**	50.01	<0.001
Deception	1.7%	**3.2%**	25.78	<0.001
Dependence	**40.4%**	22.2%	490.58	<0.001
Financial Harms	3.2%	**6.6%**	74.74	<0.001
Health Harms	**5.1%**	2.0%	95.02	<0.001
Interferes with Life	3.7%	3.8%	0.16	0.693
Interpersonal Harms	2.2%	**5.1%**	74.61	<0.001
Narrowing of Repertoire	0.8%	**2.5%**	51.24	<0.001
Patterns of Use	**22.7%**	19.6%	18.65	<0.001
Physical Signs	5.4%	**13.2%**	216.50	<0.001
Psychological Harms	5.7%	**9.5%**	61.19	<0.001
Rationalization	1.2%	1.0%	0.84	0.358
Workplace or School Harms	**1.4%**	0.6%	25.18	<0.001

Bold denotes greatest percentage response in that parent node.

**Table 5:** Unique themes nominated by people with lived experiences for specific addictions.

	Indicators	Themes	% Responses
**Alcohol**	Dependence	Not knowing when to stop	0.3%
	Physical signs	Hangovers	0.4%
		Impaired motor coordination	0.6%
		Passing out	0.3%
		Speech impairment	0.6%
**Video Games**	Patterns of use	Playing all night	0.5%
	Psychological harms	Unreality	1.9%
**Cocaine**	Physical signs	Nose issues	2.7%
**Eating**	Health harms	Diabetes	0.2%
		Digestive issues	0.1%
		High cholesterol	0.04%
	Patterns of use	Unhealthy eating habits	2.4%
		Eating without hunger	2.7%
	Psychological harms	Use as a reward	0.04%
**Sex**	Behavioral signs	Masturbation	0.8%
		Promiscuity and indiscriminate behavior	11.5%
	Interpersonal harms	Romantic problems and infidelity	5.0%
	Physical signs	Difficulty having sex	0.6%
		Erectile problems	0.5%
	Psychological harms	Lack of sexual pleasure	0.5%
		Pornography interferes with sex life	0.4%
**Shopping**	Patterns of use	Hoarding and clutter	6.8%
		Unnecessary purchases	14.7%
**Tobacco**	Dependence	Smoking despite dislike	0.2%
		Smoking in cold weather	0.9%
	Physical signs	Yellowing of fingers and nails	2.4%
**Gambling**	Patterns of use	Chasing losses	1.5%
		Re-betting winnings	0.7%
**Cannabis**	Psychological harms	Lack of motivation	2.2.%

## Supplementary Materials

The following are available online at https://www.mdpi.com/2077-0383/9/2/334/s1, Table S1: Gender differences in symptoms in first hand alcohol respondents. Table S2: Gender differences in symptoms in first hand cocaine respondents. Table S3: Gender differences in symptoms in first hand tobacco respondents. Table S4: Gender differences in symptoms in first hand cannabis respondents. Table S5: Gender differences in symptoms in first hand gambling respondents. Table S6: Gender differences in symptoms in first hand shopping respondents. Table S7: Gender differences in symptoms in first hand sex respondents. Table S8: Gender differences in symptoms in first hand work respondents. Table S9: Gender differences in symptoms in first hand video game respondents. Table S10: Gender differences in symptoms in first hand eating respondents.

## References

[1] Jacobs D.F. (1986). A general theory of addictions: A new theoretical model. J. Gambl. Behav..

[2] Orford J. (2001). Addiction as excessive appetite. Addiction.

[3] Shaffer H.J., LaPlante D.A., LaBrie R.A., Kidman R.C., Donato A.N., Stanton M.V. (2004). Toward a syndrome model of addiction: Multiple expressions, common etiology. Harv. Rev. Psychiatry.

[4] Griffiths M. (2005). A ‘components’ model of addiction within a biopsychosocial framework. J. Subst. Use.

[5] Grant J.E., Potenza M.N., Weinstein A., Gorelick D.A. (2010). Introduction to behavioral addictions. Am. J. Drug Alcohol Abus..

[6] Kim H.S., Hodgins D.C. (2018). Component model of addiction treatment: A pragmatic transdiagnostic treatment model of behavioral and substance addictions. Front. Psychiatry.

[7] Volkow N.D., Koob G.F., McLellan A.T. (2016). Neurobiologic advances from the brain disease model of addiction. N. Engl. J. Med..

[8] Konkoly Thege B., Horwood L., Slater L., Tan M.C., Hodgins D.C., Wild T.C. (2017). Relationship between interpersonal trauma exposure and addictive behaviors: A systematic review. BMC Psychiatry.

[9] Nutt D., King L.A., Saulsbury W., Blakemore C. (2007). Development of a rational scale to assess the harm of drugs of potential misuse. Lancet.

[10] American Psychiatric Association (2013). Diagnostic and Statistical Manual of Mental Disorders (DSM-5®).

[11] Reilly C., Smith N. (2013). The evolving definition of pathological gambling in the DSM-5. Natl. Cent. Responsible Gaming.

[12] Saunders J.B. (2017). Substance use and addictive disorders in DSM-5 and ICD 10 and the draft ICD 11. Curr. Opin. Psychiatry.

[13] Yücel M., Oldenhof E., Ahmed S.H., Belin D., Billieux J., Bowden-Jones H., Daglish M. (2019). A transdiagnostic dimensional approach towards a neuropsychological assessment for addiction: An international Delphi consensus study. Addiction.

[14] Fusar-Poli P., Solmi M., Brondino N., Davies C., Chae C., Politi P., McGuire P. (2019). Transdiagnostic psychiatry: A systematic review. World Psychiatry.

[15] Kim H.S., Hodgins D.C. (2019). A review of the evidence for considering gambling disorder (and other behavioral addictions) as a disorder due to addictive behaviors in the ICD-11: A focus on case-control studies. Curr. Addict. Rep..

[16] Shaffer H.J., Tom M.A., Wiley R.C., Wong M.F., Chan E.M., Cheng G.L., Lo C.K.M., Ma E.K., Wong R.H., Lee M. (2018). Using the Syndrome Model of Addiction: A preliminary consideration of psychological states and traits. Int. J. Ment. Health Addict..

[17] Blanco C., Iza M., Rodríguez-Fernández J.M., Baca-García E., Wang S., Olfson M. (2015). Probability and predictors of treatment-seeking for substance use disorders in the US. Drug Alcohol Depend..

[18] Hodgins D.C., Stea J.N., Grant J.E. (2011). Gambling disorders. Lancet.

[19] Billieux J., Schimmenti A., Khazaal Y., Maurage P., Heeren A. (2015). Are we overpathologizing everyday life? A tenable blueprint for behavioral addiction research. J. Behav. Addict..

[20] Kaptsis D., King D.L., Delfabbro P.H., Gradisar M. (2016). Withdrawal symptoms in Internet gaming disorder: A systematic review. Clin. Psychol. Rev..

[21] Kardefelt-Winther D., Heeren A., Schimmenti A., van Rooij A., Maurage P., Carras M., Edman J., Blaszczynski A., Khazaal Y., Billieux J. (2017). How can we conceptualize behavioural addiction without pathologizing common behaviours?. Addiction.

[22] Delfabbro P., Thomas A., Armstrong A. (2018). Gender differences in the presentation of observable risk indicators of problem gambling. J. Gambl. Stud..

[23] Davison C., Smith G.D., Frankel S. (1991). Lay epidemiology and the prevention paradox: The implications of coronary candidacy for health education. Sociol. Health Illn..

[24] Lovatt M., Eadie D., Meier P.S., Li J., Bauld L., Hastings G., Holmes J. (2015). Lay epidemiology and the interpretation of low-risk drinking guidelines by adults in the United Kingdom. Addiction.

[25] Schluter M.G., Hodgins D.C., Wolfe J., Wild T.C. (2018). Can one simple questionnaire assess substance-related and behavioural addiction problems? Results of a proposed new screener for community epidemiology. Addiction.

[26] Konkoly Thege B., Colman I., el-Guebaly N., Hodgins D.C., Patten S.B., Schopflocher D., Wolfe J., Wild T.C. (2015). Social judgments of behavioral versus substance-related addictions: A population-based study. Addict. Behav..

[27] Konkoly Thege B., Colman I., el-Guebaly N., Hodgins D.C., Patten S.B., Schopflocher D., Wolfe J., Wild T.C. (2015). Substance-related and behavioural addiction problems: Two surveys of Canadian adults. Addict. Res. Theory.

[28] Elo S., Kyngäs H. (2008). The qualitative content analysis process. J. Adv. Nurs..

[29] Wozniak L., Prakash M., Taylor M., Wild T.C. (2007). Everybody’s got it, but…: Situational and strategic participation in normalized HCV discourse among injection drug users in Edmonton, Canada. Int. J. Drug Policy.

[30] Gwet K.L. (2008). Computing inter-rater reliability and its variance in the presence of high agreement. Br. J. Math. Stat. Psychol..

[31] Allsopp K., Read J., Corcoran R., Kinderman P. (2019). Heterogeneity in psychiatric diagnostic classification. Psychiatry Res..

[32] Maj M. (2018). Why the clinical utility of diagnostic categories in psychiatry is intrinsically limited and how we can use new approaches to complement them. World Psychiatry.

[33] Sussman S., Lisha N., Griffiths M. (2011). Prevalence of the addictions: A problem of the majority or the minority?. Eval. Health Prof..

[34] Kasza K.A., Ambrose B.K., Conway K.P., Borek N., Taylor K., Goniewicz M.L., Cummings K.M., Sharma E., Pearson J.L., Green V.R. (2017). Tobacco-product use by adults and youths in the United States in 2013 and 2014. N. Engl. J. Med..

[35] Petry N.M., Zajac K., Ginley M.K. (2018). Behavioral addictions as mental disorders: To be or not to be?. Annu. Rev. Clin. Psychol..

[36] Peracchia S., Curcio G. (2018). Exposure to video games: Effects on sleep and on post-sleep cognitive abilities. A sistematic review of experimental evidences. Sleep Sci..

[37] De Mattos C.N., Kim H.S., Lacroix E., Requião M., Filomensky T.Z., Hodgins D.C., Tavares H. (2018). The need to consume: Hoarding as a shared psychological feature of compulsive buying and binge eating. Compr. Psychiatry.

[38] Widyanto L., Griffiths M.D., Brunsden V. (2011). A psychometric comparison of the Internet Addiction Test, the Internet-Related Problem Scale, and self-diagnosis. Cyberpsychol. Behav. Soc. Netw..

