# Supplementary material for: Transdiagnostic or Disorder Specific? Indicators of Substance and Behavioral Addictions Nominated by People with Lived Experience
Source: J Clin Med. 2020 Jan 24;9(2):334. doi: 10.3390/jcm9020334 (PMC7073953; doi:10.3390/jcm9020334)
Supplement: Supplementary file 1 [file jcm-09-00334-s001.pdf]

Supplemental Tables

Table S1. Gender differences in symptoms in first hand **alcohol** respondents

	**Males (n=301)**		**Females (n=247)**		χ^2	p
	**N**	**%**	**N**	**%**		
Behavioral Signs	34	11.3	24	9.7	0.36	0.550
Deception	20	6.6	27	10.9	3.18	0.075
Dependence	155	51.5	144	58.3	2.53	0.111
Financial Harms	11	3.7	7	2.8	0.29	0.592
Health Harms	5	1.7	5	2.0	0.10	0.752
Interferes with Life	38	12.6	34	13.8	0.16	0.694
Interpersonal Harms	19	6.3	26	10.5	3.20	0.074
Narrowing of Repertoire	6	2.0	7	2.8	0.41	0.520
Patterns of Use	127	42.2	119	48.2	1.97	0.161
Physical Signs	36	12.0	19	7.7	2.74	0.098
Psychological Harms	46	15.3	33	13.4	0.41	0.524
Rationalization	13	4.3	18	7.3	2.24	0.134
Workplace or School Harms	23	7.6	23	9.3	0.49	0.483

Table S2. Gender differences in symptoms in first hand **cocaine** respondents

	**Males (n=50)**		**Females (n=47)**		χ^2	***p***
	**N**	**%**	**N**	**%**		
Behavioral Signs	9	18.0	13	27.7	1.29	0.256
Deception	5	10.0	2	4.3	Fisher's	0.437*
Dependence	21	42.0	24	51.1	0.80	0.371
Financial Harms	12	24.0	13	27.7	0.17	0.680
Health Harms	2	4.0	0	0.0	Fisher's	0.495*
Interferes with Life	3	6.0	7	14.9	Fisher's	0.191*
Interpersonal Harms	6	12.0	6	12.8	0.013	0.909
Narrowing of Repertoire	2	4.0	1	2.1	Fisher's	1.000*
Patterns of Use	10	20.0	10	21.3	0.02	0.877
Physical Signs	5	10.0	11	23.4	3.16	0.075
Psychological Harms	15	30.0	8	17.0	2.26	0.133
Rationalization	2	4.0	0	0.0	Fisher's	0.495*
Workplace or School Harms	1	2.0	3	6.4	Fisher's	0.352*

*Fisher's Exact test was used as expected cell counts were less than 5.

Table S3. Gender differences in symptoms in first hand **tobacco** respondents

	**Males (*n*=537)**		**Females (*n*=708)**		χ^2	***p***
	**N**	**%**	**N**	**%**		
Behavioral Signs	16	3.0	13	1.8	1.76	0.185
Deception	7	1.3	8	1.1	0.08	0.781
Dependence	322	60.0	526	74.3	28.88	**<0.001****
Financial Harms	33	6.1	47	6.6	0.12	0.725
Health Harms	93	17.3	85	12.0	7.04	**0.008***
Interferes with Life	34	6.3	43	6.1	0.04	0.852
Interpersonal Harms	2	0.4	8	1.1	Fisher's	0.202
Narrowing of Repertoire	5	0.9	17	2.4	3.80	0.051
Patterns of Use	239	44.5	271	38.3	4.90	**0.027***
Physical Signs	53	9.9	39	5.5	8.49	**0.004***
Psychological Harms	29	5.4	56	7.9	3.02	0.082
Rationalization	10	1.9	9	1.3	0.71	0.400
Workplace or School Harms	9	1.7	1	0.1	Fisher's	**0.003***

* Fisher's Exact test was used as expected cell counts were less than 5.
Bold denotes significant differences

Table S4. Gender differences in symptoms in first hand **cannabis** respondents

	**Males (*n*=93)**		**Females (*n*=111)**		χ^2	***p***
	**N**	**%**	**N**	**%**		
Behavioral Signs	8	8.6	10	9.0	0.01	0.919
Deception	3	3.2	3	2.7	Fisher’s	1.000*
Dependence	44	47.3	64	57.7	2.17	0.140
Financial Harms	8	8.6	18	16.2	2.64	0.104
Health Harms	2	2.2	1	0.9	Fisher’s	0.593*
Interferes with Life	9	9.7	6	5.4	1.36	0.244
Interpersonal Harms	9	9.7	8	7.2	0.40	0.525
Narrowing of Repertoire	1	1.1	2	1.8	Fisher’s	1.000*
Patterns of Use	32	34.4	46	41.4	1.06	0.303
Physical Signs	10	10.8	12	10.8	0.00	0.989
Psychological Harms	10	10.8	30	27.0	8.50	**0.004**
Rationalization	2	2.2	3	2.7	Fisher’s	1.000*
Workplace or School Harms	5	5.4	4	3.6	Fisher’s	0.735*

*Fisher’s Exact test was used as expected cell counts were less than 5.
Bold denotes significant differences

Table S5. Gender differences in symptoms in first hand **gambling** respondents

	**Males (n=120)**		**Females (n=82)**		χ^2	***p***
	**N**	**%**	**N**	**%**		
Behavioral Signs	9	7.5	8	9.8	0.32	0.571
Deception	14	11.7	17	20.7	3.08	0.079
Dependence	45	37.5	37	45.1	1.17	0.279
Financial Harms	60	50.0	56	68.3	6.67	**0.010**
Health Harms	0.0	0.0	0.0	0.0	-	-
Interferes with Life	5	4.2	1	1.2	Fisher's	0.404*
Interpersonal Harms	5	4.2	7	8.5	Fisher's	0.233*
Narrowing of Repertoire	3	2.5	2	2.4	Fisher's	1.000*
Patterns of Use	32	26.7	21	25.6	0.03	0.867
Physical Signs	0	0.0	1	1.2	Fisher's	0.406*
Psychological Harms	15	12.5	11	13.4	0.04	0.849
Rationalization	2	1.7	1	1.2	Fisher's	1.000*
Workplace or School Harms	1	0.8	3	3.7	Fisher's	0.306*

*Fisher's Exact test was used as expected cell counts were less than 5.
Bold denotes significant differences

Table S6. Gender differences in symptoms in first hand **shopping** respondents

	**Males (n=71)**		**Females (n=195)**		χ^2	p
	**N**	**%**	**N**	**%**		
Behavioral Signs	1	1.4	4	2.1	Fisher's	1.000*
Deception	2	2.8	12	6.2	Fisher's	0.366*
Dependence	22	31.0	78	40.0	1.80	0.179
Financial Harms	44	62.0	123	63.1	0.03	0.869
Health Harms	0	0.0	0	0.0	-	-
Interferes with Life	0	0.0	8	4.1	Fisher's	0.114*
Interpersonal Harms	0	0.0	4	2.1	Fisher's	0.576*
Narrowing of Repertoire	0	0.0	2	1.0	Fisher's	1.000*
Patterns of Use	31	43.7	98	50.3	0.91	0.341
Physical Signs	0	0.0	0	0.0	-	-
Psychological Harms	6	8.5	18	9.2	0.04	0.844
Rationalization	2	2.8	6	3.1	0.01	0.913
Workplace or School Harms	0	0.0	1	0.5	Fisher's	1.000*

*Fisher's Exact test was used as expected cell counts were less than 5.

Table S7. Gender differences in symptoms in first hand **sex** respondents

	**Males (*n*=223)**		**Females (*n*=88)**		χ^2	***p***
	**N**	**%**	**N**	**%**		
Behavioral Signs	115	51.6	47	53.4	0.09	0.770
Deception	16	7.2	1	1.1	4.45	**0.035**
Dependence	114	51.1	38	43.2	1.59	0.207
Financial Harms	5	2.2	1	1.1	Fisher's	1.000*
Health Harms	3	1.3	3	3.4	Fisher's	0.356*
Interferes with Life	16	7.2	6	6.8	0.01	0.912
Interpersonal Harms	52	23.3	17	19.3	0.59	0.444
Narrowing of Repertoire	8	3.6	3	3.4	Fisher's	1.000*
Patterns of Use	42	18.8	16	18.2	0.02	0.894
Physical Signs	6	2.7	5	5.7	Fisher's	0.303*
Psychological Harms	22	9.9	16	18.2	4.07	**0.044***
Rationalization	5	2.2	1	1.1	Fisher's	1.000
Workplace or School Harms	5	2.2	3	3.4	Fisher's	0.692*

*Fisher's Exact test was used as expected cell counts were less than 5.
Bold denotes significant differences.

Table S8. Gender differences in symptoms in first hand **work** respondents

	**Males (*n*=435)**		**Females (*n*=444)**		χ^2	***p***
	**N**	**%**	**N**	**%**		
Behavioral Signs	27	6.2	42	9.5	3.21	0.073
Deception	1	0.2	1	0.2	0.00	0.988
Dependence	159	36.6	180	40.5	1.48	0.224
Financial Harms	5	1.1	1	0.2	Fisher’s	0.120*
Health Harms	15	3.4	22	5.0	1.24	0.266
Interferes with Life	51	11.7	67	15.1	2.14	0.143
Interpersonal Harms	78	17.9	74	16.7	0.25	0.620
Narrowing of Repertoire	39	9.0	58	13.1	3.76	0.053
Patterns of Use	144	33.1	155	34.9	0.319	0.572
Physical Signs	0	0.0	0	0.0	-	-
Psychological Harms	139	32.0	196	44.1	13.84	**<0.001**
Rationalization	3	0.7	2	0.5	Fisher’s	0.684*
Workplace or School Harms	0	0.0	0	0.0	-	-

*Fisher’s Exact test was used as expected cell counts were less than 5.
Bold denotes significant differences

Table S9. Gender differences in symptoms in first hand **video game** respondents

	**Males (*n*=118)**		**Females (*n*=73)**		χ^2	***p***
	**N**	**%**	**N**	**%**		
Behavioral Signs	6	5.1	3	4.1	Fisher's	1.000*
Deception	2	1.7	1	1.4	Fisher's	1.000*
Dependence	53	44.9	41	56.2	2.28	0.131
Financial Harms	7	5.9	3	4.1	Fisher's	0.744*
Health Harms	0	0.0	1	1.4	Fisher's	0.382*
Interferes with Life	28	23.7	17	23.3	0.01	0.944
Interpersonal Harms	31	26.3	16	21.9	0.461	0.497
Narrowing of Repertoire	12	10.2	13	17.8	Fisher's	0.184*
Patterns of Use	42	35.6	24	32.9	0.15	0.701
Physical Signs	18	15.3	13	17.8	0.22	0.642
Psychological Harms	12	10.2	13	17.8	2.31	0.128
Rationalization	0	0.0	1	1.4	Fisher's	0.382*
Workplace or School Harms	9	7.6	5	6.8	0.04	0.841

*Fisher's Exact test was used as expected cell counts were less than 5.

Table S10. Gender differences in symptoms in first hand **eating** respondents

	**Males (*n*=282)**		**Females (*n*=753)**		χ^2	***p***
	**N**	**%**	**N**	**%**		
Behavioral Signs	15	5.3	47	6.2	0.31	0.578
Deception	18	6.4	91	12.1	7.08	**0.008**
Dependence	79	28.0	283	37.6	8.26	**0.004**
Financial Harms	2	0.7	4	0.5	Fisher's	0.666*
Health Harms	24	8.5	53	7.1	0.65	0.422
Interferes with Life	5	1.8	23	3.1	1.28	0.258
Interpersonal Harms	0	0.0	18	2.4	Fisher's	**0.006***
Narrowing of Repertoire	5	1.8	12	1.6	Fisher's	0.789*
Patterns of Use	117	41.5	300	39.8	0.232	0.630
Physical Signs	180	63.8	376	49.9	15.94	**<0.001**
Psychological Harms	13	4.6	61	8.1	3.77	0.052
Rationalization	9	3.2	30	4.0	0.36	0.551
Workplace or School Harms	1	0.4	1	0.1	Fisher's	0.471*

*Fisher's Exact test was used as expected cell counts were less than 5.
Bold denotes significant differences.
